# Comparisons of Clinical Characteristics and Surgical Outcomes of Epiretinal Membrane Foveoschisis to Typical Epiretinal Membrane

**DOI:** 10.3390/jcm12124009

**Published:** 2023-06-12

**Authors:** Taku Sasaki, Yoshitsugu Matsui, Kumiko Kato, Shinichiro Chujo, Satoshi Maeda, Atsuta Ozaki, Kengo Ikesugi, Masahiko Sugimoto, Hisashi Matsubara, Mineo Kondo

**Affiliations:** 1 Department of Ophthalmology, Mie University Graduate School of Medicine, Tsu 514-8507, Japan; caesium1860@gmail.com (T.S.); footboyslim366@gmail.com (Y.M.); k-kato@med.mie-u.ac.jp (K.K.); shinchujo21@gmail.com (S.C.); tokingy04@gmail.com (S.M.); atsuta3527@gmail.com (A.O.); ikesugi@med.mie-u.ac.jp (K.I.); sugmochi92@gmail.com (M.S.); hmatsu@med.mie-u.ac.jp (H.M.); 2 Department of Ophthalmology and Visual Sciences, Yamagata University Faculty of Medicine, Yamagata 990-8585, Japan

**Keywords:** epiretinal membrane, epiretinal membrane foveoschisis, sex, vitrectomy

## Abstract

Epiretinal membrane (ERM) foveoschisis is a recently proposed clinical entity. The purpose of this study was to compare the clinical characteristics and surgical outcomes of eyes with ERM foveoschisis to those of typical ERM. The medical records of all patients with ERM-related disorders examined between 2011 and 2020 were reviewed. ERM foveoschisis was defined by the clinical criteria proposed by an international panel of experts on ERMs. The background factors, clinical characteristics, and surgical outcomes of ERM foveoschisis were compared to those of typical ERM. Forty eyes with ERM foveoschisis were compared to 333 eyes with typical ERM. The percentage of women was significantly higher in the ERM foveoschisis group (92.5%) than in the typical ERM group (48.9%, *p* < 0.001). The central macular thickness (CMT) was significantly thinner in the ERM foveoschisis group (340 ± 110 μm) than in the typical ERM groups (476 ± 111 μm, *p* < 0.01). The degree of improvement in the best-corrected visual acuity (BCVA) three months after the surgery did not differ between the two groups (*p* = 0.59). These results suggest that the ERM foveoschisis is more likely to occur in women and that the prognosis after surgery is comparable to typical ERM.

## 1. Introduction

In 2020, an international panel of vitreoretinal experts tried to arrive at a consensus on the clinical characteristics that can be used to diagnose a lamellar macular hole (LMH) and similar conditions based on optical coherence tomographic (OCT) images [1]. In the end, the panel reached a consensus on the definition of three clinical entities an LMH, an epiretinal membrane (ERM) foveoschisis, and a macular pseudohole (MPH). 

Of these three entities, the ERM foveoschisis was a relatively new disease entity, and the expert panel defined an ERM foveoschisis by the OCT findings with two mandatory and three optional diagnostic criteria. The two mandatory criteria were a contractile ERM and a foveoschisis at the level of Henle’s fiber layer. The three optional criteria were the presence of microcystoid spaces in the inner nuclear layer, retinal thickening, and retinal wrinkling. ERM foveoschisis was previously referred to as a “tractional type of LMH” to distinguish it from a “degenerative LMH” [2,3,4,5,6], although other names have been used [7,8,9]. Recently, several studies have reported on the clinical features [10,11,12] and postoperative changes in eyes with ERM foveoschisis [13,14,15,16].

ERM foveoschisis and typical ERM have similar characteristics, including the presence of a fibrous membrane on the surface of the retina that can contract, causing changes in the retinal morphology. These changes result in blurred vision and metamorphopsia. However, it has not been determined why the schisis developed in Henle’s fiber layer in eyes with ERM foveoschisis and not in eyes with typical ERMs. We reasoned that the comparisons of background factors and clinical characteristics of these two patient groups might provide clues that could help in understanding the pathophysiology of why the schisis occurs in Henle’s layer of the retina of patients with ERM foveoschisis. In addition, the results could contribute to better treatment for patients with ERM foveoschisis.

Thus, the purpose of this study was to compare the clinical characteristics of eyes with ERM foveoschisis to those with typical ERM without schisis. We also considered whether there were differences in the short-term outcomes after vitrectomy between eyes with ERM foveoschisis to eyes with typical ERM. 

## 2. Materials and Methods

### 2.1. Study Design and Approval

This was a retrospective study of the medical records of patients examined at the Mie University Hospital of Japan. The study protocol was approved by the Ethics Committee of the Mie University Hospital (#H2021-016). Written informed consent was not obtained from the subjects because of the retrospective nature of this study. Instead, a home page was created with information on the purpose of this study for the subjects to read. We emphasized in the text that any subject could opt out of the study at any time by telephone, fax, or e-mail. The data extracted for this study were anonymized before they were examined. The procedures used conformed to the tenets of the Declaration of Helsinki of the World Medical Association.

### 2.2. Subjects

The search function of the electronic medical records program was used to extract all patients with ERM-related diseases who had been examined in the Mie University Hospital between August 2011 and December 2020. When searching, all patients registered with the following disease names were extracted: Epiretinal membrane (ERM), epimacular membrane, macular pucker, cellophane maculopathy, macular pseudohole (MPH), and lamellar macular hole (LMH). If a patient had an ERM-related disorder in both eyes, only the eye with the earlier onset was used. The diagnosis of the disease was made by two retinal specialists (TS, MK) using fundus photographs and OCT findings according to the definition described in the next paragraph. 

Patients with a history of advanced glaucoma or other retinal disease such as diabetic retinopathy, retinal vein occlusion, or age-related macular degeneration were excluded. We also excluded high myopic eyes, which had either an axial length ≥26.5 mm or a refractive error (spherical equivalent) of ≥−6.00 diopters (D). These exclusion criteria were used because we did not measure the axial length for all eyes. 

### 2.3. Definition of Subtypes of ERM-Related Diseases

**Figure 1:**
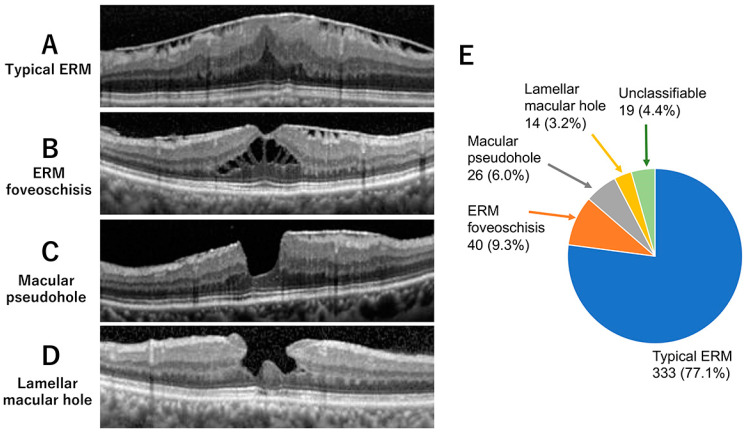
Representative optical coherence tomographic (OCT) B-scan images of an eye with typical epiretinal membrane (ERM) (**A**), ERM foveoschisis (**B**), macular pseudohole (MPH, (**C**)), and lamellar macular hole (LMH, (**D**)). (**E**) Number and percentage of eyes of each subtype among 432 eyes with ERM-related diseases.

Typical ERM was diagnosed by the presence of hyperreflective fibrocellular proliferations on the surface of the internal limiting membrane (Figure 1A). Contracture of an ERM is often associated with a wrinkling of the underlying retina, flattening of the foveal pit, and thickening of the retina [2,17,18,19]. 

ERM foveoschisis was diagnosed by the presence of a contractile ERM and the presence of a foveoschisis at the level of Henle’s fiber layer (Figure 1B). Other optional criteria included the presence of microcystoid spaces in the inner nuclear layer, retinal thickening, and retinal wrinkling [1,2,3,8].

An MPH was defined by the presence of a foveal sparing ERM, retinal thickening, and a vertical and steepened foveal profile (Figure 1C) [1,2,3,8,18,19,20,21]. Other minor criteria were the presence of microcystoid spaces in the inner nuclear layer and near the central foveal thickness.

An LMH was diagnosed by the presence of an irregular foveal contour, i.e., abnormal, non-linear shape of the foveal pit contour, foveal cavity with undermined edges, and presence of at least one other sign evoking a loss of foveal tissue (Figure 1D). Other minor criteria were epiretinal proliferation, foveal bump, and disruption of the ellipsoid zone [1,2,8,18,19,20,21].

The classifications in our cases were made by two retinal specialists (TS and MK) independently. If the two decisions did not agree, or if the retina had two or three features of subtypes, then the eye was classified as “unclassifiable”.

### 2.4. Ophthalmological Examinations

The best-corrected visual acuity (BCVA) was measured with a standard Japanese decimal visual acuity chart at 5 m. The decimal values were converted to the logarithm of the minimal angle of resolution (logMAR) units for the statistical analyses. 

Optical coherence tomography (OCT) was performed with a spectral-domain OCT instrument (Spectralis HRA + OCT, Heidelberg Engineering, Vista, CA, USA). Horizontal and vertical B-scan images that spanned 30° or approximately 9 mm were recorded in the ‘high-resolution mode’, and 50 B-scan images were averaged. The central macular thickness (CMT) and the average thickness within a 1-mm diameter of the central macular area were also measured. The axial length was measured by an optical biometer (OA 2000^®^; Tomey, Nagoya, Japan).

### 2.5. Surgical Procedures

Surgery was performed when the decimal BCVA decreased to approximately ≤0.8 or when the patient requested surgery due to blurred vision or metamorphopsia, even though the decimal BCVA was better than 0.8. Vitrectomy was performed through a 25 or 27-gauge pars plana microincision by one of six surgeons (TS, YM, SC, MS, HM, and MK). Triamcinolone acetonide was used intraoperatively in all eyes to increase the visibility of the vitreous and posterior hyaloid. The ERM and the ILM were peeled in the macular area using forceps after the ILM was stained with 0.25 mg/mL brilliant blue G solution (Coomassie BBG 250; Sigma-Aldrich, St. Louis, MO, USA). Cataract extraction with implantation of a posterior chamber intraocular lens was performed at the same time as the pars plana vitrectomy in all eyes with a cataract. 

### 2.6. Statistical Analyses

After confirming that the data were normally distributed, unpaired *t*-tests were used to determine whether the values of continuous variables in the two groups were significantly different. Chi-square tests were used to compare the ratios of various clinical findings between the two groups. Paired *t*-tests were used to determine whether the values of the BCVA or CMT between the baseline and at 3 months after the surgery were significantly different. The results were considered statistically significant when *p* < 0.05. Analyses were performed with SPSS software, version 20 (IBM SPSS Statistics 20, IBM Corp., New York, NY, USA).

## 3. Results

### 3.1. Incidence of ERM Foveoschisis

A search of the electronic medical record extracted 526 patients with ERM-related disorders. After the confirmation by two retina specialists (TS, MK) using fundus photographs and OCT images, 135 eyes were excluded due to a misdiagnosis or missing data. The results of the classification for the remaining 432 eyes are shown in Figure 1E. Of the 432 eyes with ERM-related disorders, 333 eyes (77.1%) were typical ERM, 40 eyes (9.3%) were ERM foveoschisis, 26 eyes (6.0%) were MPH, and 14 eyes (3.2%) were LMH. Nineteen eyes (4.4%) were judged to be unclassifiable or to have features of two different subtypes.

### 3.2. Comparison of Clinical Characteristics

We compared the clinical characteristics of patients with ERM foveoschisis to those with typical ERM (Table 1). There was no significant difference in the mean age (*p* = 0.26) and the mean axial length between the two groups (*p* = 0.54) even though the axial length had been measured in only 75.0% of the ERM foveoschisis and 76.0% of typical ERM group.

Interestingly, the percentage of women was significantly higher in the ERM foveoschisis group (92.5%) than in the typical ERM group (48.9%; *p* < 0.01). There was no significant difference in the BCVA between the two groups (*p* = 0.34).

### 3.3. Comparisons of OCT Findings

**Figure 2:**
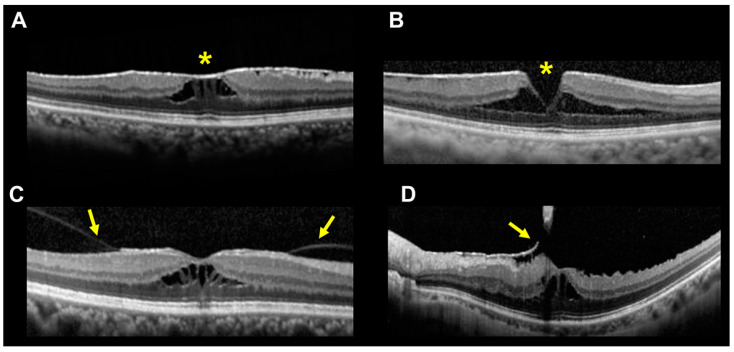
OCT B-scan images of eyes with an ERM foveoschisis. (**A**) Representative OCT B-scan images of ERM foveoschisis with presence of fibrous membrane on the retinal surface at the fovea (yellow asterisk). (**B**) Representative OCT B-scan images of ERM foveoschisis with absence of fibrous membrane at the fovea (yellow asterisk). (**C**,**D**) Two representative OCT B-scan images of ERM foveoschisis with vitreous adhesion to the macula. Yellow arrows indicate the posterior vitreous membrane with adhesion to the macula.

Next, we compared the OCT findings between the two groups (Table 2). The CMT of the ERM foveoschisis group (340 ± 110 µm) was significantly thinner than that of the typical ERM group (476 ± 111 µm, *p* < 0.01). We also determined that the percentage of eyes with an absence of fibrous membrane at the fovea centralis was significantly higher in the ERM foveoschisis group (55.0%) than in the typical ERM group (9.9%, *p* < 0.01). Representative OCT images of cases of ERM foveoschisis with (Figure 2A) and without (Figure 2B) fibrous membrane at the area centralis are shown in Figure 2. 

The percentage of eyes with a continuous ellipsoid zone (EZ) within 6 mm of the macula was significantly higher in the ERM foveoschisis group (82.5%) than in the typical ERM group (62.2%, *p* = 0.01). The presence of vitreous adhesions to the macula was higher in the ERM foveoschisis group (15.0%) than in the typical ERM group (7.2%), but this difference was not statistically significant (*p* = 0.09). Two representative OCT images of cases of ERM foveoschisis associated with vitreous adhesions to the macula are shown in Figure 2C,D.

### 3.4. Comparison of Short-Term Surgical Outcomes

**Figure 3:**
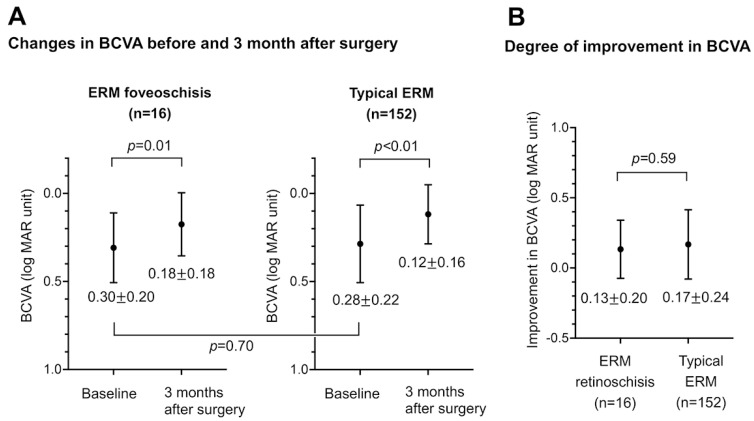
(**A**) Changes in the BCVA (logMAR units) before and three months after the surgery in the ERM foveoschisis group (n = 16, left panel) and the typical ERM group (n = 152, right panel). Baseline BCVA did not differ significantly between the two groups (*p* = 0.70). (**B**) Comparison of the degree of improvement in BCVA 3 months after the surgery between two groups.

Finally, we compared the short-term surgical outcomes between the two groups. Vitrectomy was performed on 16 of 40 eyes with ERM foveoschisis and 153 of 333 eyes with typical ERM. Cataract extraction with posterior chamber intraocular lens implantation was performed at the same time as the pars plana vitrectomy in 16 eyes with ERM foveoschisis and 152 eyes with typical ERM. We then compared the changes in the BCVA and CMT between these 16 eyes with ERM foveoschisis and 152 eyes with typical ERM. The BCVA (logMAR units) before and 3 months after the surgery for the two groups are shown in Figure 3A. The baseline BCVA did not differ significantly between the two groups (*p* = 0.70). We found that both groups had significant improvements in the BCVA at three months after the surgery (*p* < 0.05 for both), and there was no significant difference in the degree of improvement in the BCVA between the two groups (*p* = 0.59, Figure 3B).

**Figure 4:**
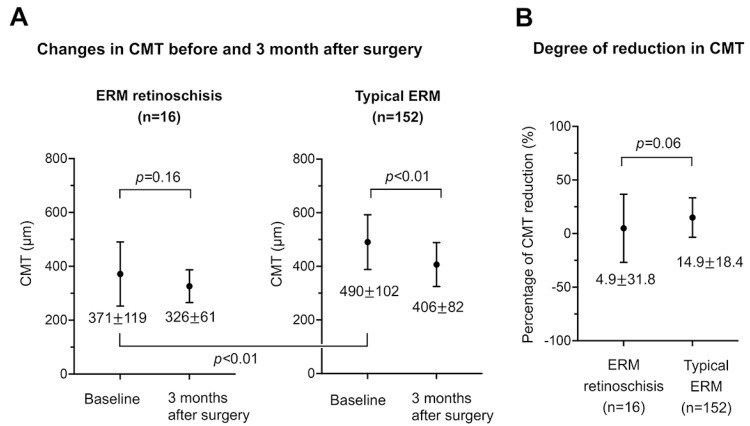
Central macular thickness (CMT) in the ERM foveoschisis and the typical ERM eyes. (**A**) Changes in the central macular thickness (CMT) before and three months after the surgery for the ERM foveoschisis group (n = 16, left panel) and the typical ERM group (n = 152, right panel). The baseline CMT was significantly thinner in the ERM foveoschisis group than in the typical ERM group (*p* < 0.01). (**B**) Comparison of the percentage reduction in the CMT three months after the surgery between two groups.

The CMTs before and three months after the surgery for the two groups are shown in Figure 4A. The baseline CMT was significantly thinner in the ERM retinoschisis group than in the typical ERM group (*p* < 0.01). Although there was a significant reduction in the CMT three months after the surgery in the typical ERM group (*p* < 0.01), the reduction was not significant in the ERM retinoschisis group (*p* = 0.16). Because the baseline CMT differed significantly between the two groups, we compared the percentage of reduction in the CMT at three months. The percentage reduction of the CMT at three months was calculated as follows: CMT reduction (%) = (baseline CMT − CMT at three months)/baseline CMT × 100. The results indicate that the ERM retinoschisis group had a marginally significantly smaller percentage of CMT reduction than the typical ERM group (*p* = 0.06).

**Figure 5:**
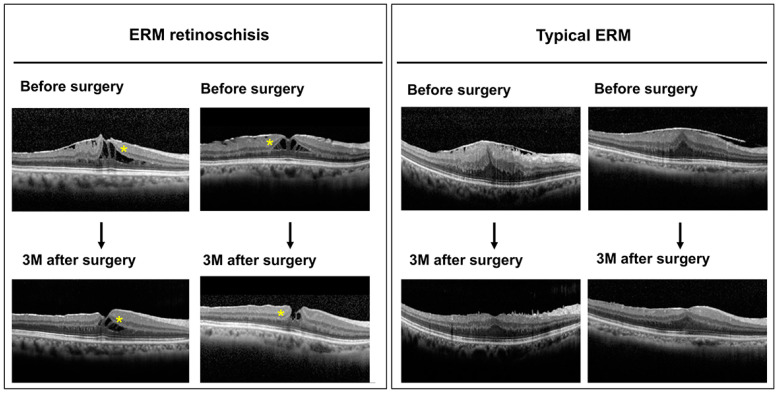
Two representative horizontal scan OCT images before and three months after the vitrectomy for the ERM foveoschisis group and typical ERG group. The postoperative CMT reduction is more apparent in the typical ERG group. ERM foveoschisis group also showed thinner CMT postoperatively, but the reduction of CMT was less evident. In the ERM foveoschisis group, we also noticed that the space of retinoschisis became smaller three months after the surgery (yellow asteriks).

Two representative horizontal OCT images before and three months after the vitrectomy for the two groups are shown in Figure 5. The postoperative CMT reduction was quite obvious in eyes with typical ERM, but not as evident in eyes with ERM retinoschisis. In eyes with ERM retinoschisis, we also found that the space of foveoschisis appeared to become narrower three months after the surgery (yellow asterisks), as reported by earlier studies [6,14,15,16].

## 4. Discussion

Our results show that the incidence of ERM foveoschisis was 9.3% among all ERM-related diseases, which was more common than that for LMH (6.0%) and MPH (3.2%, Figure 1). This indicated that ERM retinoschisis is not an uncommon subtype of ERM-related diseases. The incidence of ERM foveoschisis was comparable to that reported by Hetzel et al. at 6.7% [12], but it was higher than the 3.1% reported by Lam et al. [15]. The reason for this difference may be that Lam et al. analyzed only patients with ERM-related diseases who underwent vitrectomy [15].

The interesting result on the clinical characteristics of patients with ERM retinoschisis was the very high incidence of women. While the men-to-women ratio was almost equal in typical ERM, 92.5% of ERM retinoschisis patients were women (Table 1). Initially, we suspected that the reason for this high predominance of women might be that we included eyes with myopic maculoschisis, which is more common in Asian women [22]. However, we excluded patients with myopia ≥−6.0 D or an axial length of ≥26.5 mm. Furthermore, the axial length of eyes with ERM retinoschisis was not significantly different from those of eyes with typical ERM (Table 1). Therefore, we conclude that it was unlikely that the eyes with myopic maculoschisis were included in our group of eyes with ERM foveoschisis.

The higher incidence of ERM foveoschisis in women has been reported earlier. The percentage of women with ERM foveoschisis was reported to be 85.7% by Yeo et al. in Korea [6], 64.7% by Lam et al. in France [15], 85.7% by Photcharapongsakul et al. in Thailand [14], and 72.2% by Hetzel et al. in Germany [12]. Thus, the predominance of women in the ERM foveoschisis group is highly reliable. It is also interesting to note that all of the three studies on Asians, including our study, have shown that women are more than 80% of the patients with ERM foveoschisis [6,14]. Further international collaborative studies are needed to determine the mechanism for this disproportionate incidence in women.

Our results also show that eyes with ERM foveoschisis had thinner CMT and had a higher percentage of eyes with an absence of an ERM at the fovea centralis than eyes with typical ERM (Table 2). The lack of fibrous membrane at the fovea centralis was associated with a steep depression of the fovea [1,2,3,8]. This type of fovea was called the “open-type” of ERM foveoschisis by Hetzel et al. [12]. This can be one of the reasons why the CMT was thinner in the ERM foveoschisis than in the eyes with typical ERM.

The pathophysiology of why the schisis occurs in Henle’s layer of the retina of patients with ERM foveoschisis is not clearly understood. Gaudric et al. suggested that the cleavage of the foveal pit edge may result from asymmetrical tangential traction of the ERM between multiple epicenters of contraction [8]. They also showed that these epicenters are well highlighted with en-face OCT images [8]. If this was the case, it is reasonable to hypothesize that the foveoschisis may be more likely to form in women by such asymmetrical tangential traction.

Finally, we compared the short-term surgical outcomes between eyes with ERM foveoschisis and eyes with typical ERM. It was difficult to compare the changes in the CMT after the surgery because the baseline CMTs were significantly different between the two groups. The percentage of CMT reduction rate tended to be lower in the eyes with ERM retinoschisis than in eyes with typical ERM, but this difference may be because the baseline CMT was not so thick in the ERM foveoschisis group. 

For the BCVA, whose baseline values were comparable in the two groups, there was no significant difference in the degree of improvement at three months after the surgery. Recent studies on the visual acuity after the surgery in eyes with ERM foveoschisis also reported a significant improvement [8,13,14,15]. Our results combined with these reports suggest that the improvement of BCVA is expected in eyes with ERM foveoaschisis as well as the eyes with typical ERM.

There are several limitations to this study. The first limitation is the low number of eyes with ERM foveoschisis. A larger sample size collected by a multicenter study would be needed to study the clinical characteristics and surgical outcomes in more detail. The second limitation is the short postoperative follow-up period of three months. Because of the slow recovery of function and structure after the surgery, examinations at six months and one year are also needed. The third limitation is that most of the patients who underwent vitrectomy also underwent cataract surgery at the same time. Therefore, it is unclear to what extent the vitrectomy alone improved the visual acuity. The fourth limitation is that the decimal values of BCVA were converted to logMAR units in our study. It should be recognized that such conversions can lead to overestimation of its true value, especially for lower acuities [23]. The last limitation is that we could not determine how many eyes had PVD accurately. Initially, we tried to determine how many patients had PVD using the 9 mm vertical and horizontal scans of SD-OCT retrospectively. At the same time, we also referred to the findings of the presence or absence of PVD by fundus examinations in the medical records. The results show that the presence of a PVD was significantly lower in the ERM foveoschisis group (87.5%, 35/40 eyes) than in the typical ERM group (97.3%, 324/333 eyes, *p* < 0.01). This supports our suggestion that vitreomacular adhesion may be associated with the development of the foveoschisis. However, such retrospective methods of examining only a 9-mm length SD-OCT image and medical records for the presence of PVD were considered to be inadequate. Further prospective studies using a combination of SS-OCT with longer scan lengths, echography, and fundus observations are needed to determine accurately how many eyes had PVD. 

## 5. Conclusions

We found that the percentage of women was significantly higher in ERM foveoschisis than in the typical ERM. We also noted that the prognosis of ERM foveoschisis after the surgery is comparable to that of typical ERM.

## Figures and Tables

**Table 1:** Comparison of clinical characteristics between typical ERM and ERM foveoschisis.

	ERM Foveoschisis	Typical ERM	*p*-Value
Number of eyes/subjects	40/40	333/333	
Age, mean ± SD (range), years	69.9 ± 6.8	71.1 ± 8.4	*p* = 0.26
Sex			*p* < 0.01 **
Men, number of eyes (%)	3 (7.5%)	170 (51.1%)	
Women, number of eyes (%)	37 (92.5%)	163 (48.9%)	
Axial length, mm (number of eyes)	23.7 ± 1.1 (30)	23.6 ± 1.1 (253)	*p* = 0.54
Best-corrected visual acuity, mean ± SD, logMAR	0.23 ± 0.21	0.27 ± 0.25	*p* = 0.34

SD, standard deviation; logMAR, logarithmic minimum angle of resolution. Unpaired *t*-tests were used to determine if the age, axial length, and BCVA were significantly different between the two groups. Chi-square test was used to determine the significance of the difference in the ratio of women to men between the two groups. ** *p* < 0.01.

**Table 2:** Comparison of OCT findings between typical ERM and ERM foveoschisis.

	ERM Foveoschisis	Typical ERM	*p*-Value
Number of eyes/subjects	40/40	333/333	
Central macular thickness (CMT), mean ± SD, µm	340 ± 110	476 ± 111	*p* < 0.01 **
Absence of ERM at fovea centralis, number of eyes (%)	22 (55.0%)	33 (9.9%)	*p* < 0.01 **
Continuity of ellipsoid zone number of eyes (%)	33 (82.5%)	207 (62.2%)	*p =* 0.01 *
Presence of vitreous adhesion, number of eyes (%)	6 (15.0%)	24 (7.2%)	*p* = 0.09

SD, standard deviation; Paired *t*-tests were used to determine if the CMT were significantly different between the two groups. Chi-square test was used to compare the ratio between the two groups. * *p* < 0.05. ** *p* < 0.01.

## Data Availability

All data generated or analyzed during this study are included in this published article.
